# A Dual Bioassay for Evaluation of Embryotoxicity and Acute Toxicity of Common Solvents and Surfactants in *Artemia salina*

**DOI:** 10.3390/toxics13060442

**Published:** 2025-05-27

**Authors:** Iulia Ioana Olaru, Octavian Tudorel Olaru, Dragos Paul Mihai, Cerasela Elena Gird, Anca Zanfirescu, Rica Boscencu, Emanuela-Alice Luta, Corina Andrei, George Mihai Nitulescu

**Affiliations:** Faculty of Pharmacy, “Carol Davila” University of Medicine and Pharmacy, Traian Vuia 6, 020956 Bucharest, Romania; iulia.radulescu@drd.umfcd.ro (I.I.O.); cerasela.gird@umfcd.ro (C.E.G.); anca.zanfirescu@umfcd.ro (A.Z.); rica.boscencu@umfcd.ro (R.B.); emanuela.luta@umfcd.ro (E.-A.L.); corina.andrei@umfcd.ro (C.A.); george.nitulescu@umfcd.ro (G.M.N.)

**Keywords:** *Artemia salina*, toxicity evaluation, hatching test, solvent toxicity

## Abstract

This study evaluated the acute and developmental toxicity of selected hydrotropes, co-solvents, and surfactants commonly used in pharmaceutical and cosmetic formulations, using *Artemia salina* as a model organism. Two bioassays were employed: a lethality test and a hatching inhibition test. Compounds such as sodium lauryl sulfate (LC_50_ < 0.05%), sodium xylenesulfonate (LC_50_ = 0.79%), sodium p-toluensulfonate (LC_50_ = 0.21%), *N*,*N*-dimethylbenzamide (LC_50_ < 0.05%), and *N*,*N*-diethylnicotinamide (LC_50_ = 0.05%) exhibited high toxicity at 48 h, inducing significant mortality and strong inhibition of hatching. Glycerin, propylene glycol, and dimethyl sulfoxide showed low toxicity across all concentrations. Lethal concentration values confirmed the high toxicity of sodium xylenesulfonate and *N*,*N*-dimethylbenzamide, with moderate effects observed for other compounds. The hatching inhibition test proved more sensitive than the lethality test, enabling the detection of embryotoxicity and developmental delays. Although more laborious, it provided detailed information into how the tested substances influenced developmental stage progression. Hierarchical clustering analysis grouped the substances based on toxicity patterns and clearly discriminated highly toxic surfactants from low-toxicity solvents. The results demonstrated that combining both bioassays offers a more comprehensive evaluation of toxicity, with the hatching test being particularly useful for identifying early developmental effects not evident in lethality testing alone.

## 1. Introduction

An increasing number of new active pharmaceutical ingredients (APIs) exhibit highly lipophilic characteristics, leading to poor aqueous solubility and limited oral bioavailability [1,2]. The Biopharmaceutical Classification System (BCS) categorizes drugs based on their solubility and intestinal permeability [3]. BCS Class II compounds are poorly water-soluble but possess high permeability; for these, dissolution in gastrointestinal fluids is the rate-limiting step for absorption [4]. To overcome this barrier, formulation strategies often involve the use of solubilizers and wetting agents that enhance solubility in physiological media. Techniques such as cyclodextrin complexation, nanoparticle formation, and the development of solid solutions via hot-melt extrusion have shown promising results in increasing the apparent solubility and, consequently, the bioavailability of BCS Class II drugs [5,6].

In in vitro testing, evaluation of a drug candidate is often limited by its poor solubility, necessitating the use of solubilizers. However, to ensure the reliability of the results, it is essential to validate that the solubilizer does not interfere with the test system. While interactions between formulation parameters can be systematically validated in analytical methods using established protocols, tests involving living cells or organisms present greater complexity [7,8]. In such biological assays, solubilizers may exert unintended toxic, stimulatory, or inhibitory effects, potentially confounding the interpretation of results. Therefore, careful method evaluation, including compatibility testing of solubilizers with the biological system, is crucial to discriminate the true effects of the active pharmaceutical ingredient from those of the vehicles [9].

The increasing use of solubilizers in drug development and formulation highlights the need for thorough ecotoxicological and pharmaco-toxicological pre-screening, particularly when such substances are applied in marine or aquatic toxicity assays [10]. Many solubilizers, although effective in enhancing drug solubility, may exhibit toxic effects on aquatic organisms, thereby posing environmental risks if discharged or disposed of improperly [11].

*Artemia salina* (brine shrimp) serves as a valuable model organism for toxicity screening [12,13]. Due to its high sensitivity to a wide range of chemical agents, ease of obtaining from cysts, low cost, and ecological relevance, *Artemia* is widely used in preliminary assessments of the cytotoxicity of chemical compounds, plant extracts, and environmental impact [12,14]. Its transparent body allows for direct observation of physiological changes, and its role as a representative of marine zooplankton makes it a suitable indicator for evaluating the potential hazards of pharmaceutical substances in aquatic ecosystems. Moreover, the brine shrimp lethality assay offers a simple yet informative tool for assessing both pharmacological and ecotoxicological profiles of drug candidates during early-stage development [15]. Using *Artemia* as a preliminary screening tool can help prioritize substances for further testing, potentially reducing the number of vertebrate animals used in subsequent stages of toxicity assessment and aligning with the principles of the 3Rs (Replacement, Reduction, Refinement) [12,16].

When evaluating the toxicity of pharmaceutical compounds or excipients such as solubilizers, both the brine shrimp lethality test and the *Artemia salina* hatching test are employed due to their simplicity and relevance. However, these assays differ in sensitivity, methodology, and the type of toxicological information they provide [17].

The brine shrimp lethality test is an acute toxicity assay that determines the mortality rate of *Artemia* nauplii after exposure to a test compound over a fixed period, typically 24 or 48 h. It is rapid, cost-effective, and suitable for high-throughput screening. The test evaluates the direct cytotoxic effects of substances on already hatched nauplii, making it particularly useful for assessing acute toxicity and comparing the relative potencies of different compounds [18,19]. The hatching test focuses on the ability of *Artemia* cysts to hatch under exposure to test substances. This method provides insight into sub-lethal and developmental toxicity by examining how a compound interferes with embryonic development and hatching success. It may detect toxic effects that are not immediately lethal but can impair organism viability or development. Together, the brine shrimp lethality test and hatching test could offer complementary data regarding acute toxicity and developmental and embryotoxic effects [20].

In the present study, a diverse panel of substances were selected to evaluate their applicability as solubilizers and their cytotoxicity profile in the brine shrimp lethality test and *Artemia* hatching test, two screening methods that use *Artemia salina* as a testing organism. The tested compounds include commonly used solubilizers and surfactants, as well as small molecular vehicles with varying degrees of hydrophilicity and lipophilicity. These substances were sodium xylenesulfonate (SXS), sodium benzenesulfonate (SBS), sodium p-toluenesulfonate (PTS), and sodium benzene-1,3-disulfonate (SBDS), all of which are aromatic sulfonates with detergent-like properties [21,22]. Nitrogen-containing solubilizers such as N,N-dimethylbenzamide (DMBA), N,N-diethylnicotinamide (DENA), N,N-dimethylurea (DMU), and urea were also included for their known hydrogen bonding and polarity-modifying characteristics [23]. Solvents such as dimethylformamide (DMF) and dimethyl sulfoxide (DMSO) were tested due to their high solubilizing power [24] and common use in bioassays [25]. Additionally, the nonionic surfactants Tween 20 and Tween 80 (Polysorbates 20 and 80), sodium lauryl sulfate (SLS), and humectants such as glycerol (GLY) and propylene glycol (PDO) were evaluated. These agents are frequently employed in oral and parenteral formulations to enhance drug solubility and stability [26], and occasionally in bioassays [27,28]. A control group without additives was also included for comparison. The selection of these compounds allows for a comprehensive assessment of both solubilizing efficiency and biocompatibility in the test system. The hypothesis proposed is that these substances may serve as additives to enhance the applicability of *Artemia* tests, particularly for compounds with low solubility.

## 2. Materials and Methods

### 2.1. Chemicals

SXS (CAS 1300-72-7), SBS (CAS 515-42-4), PTS (CAS 657-84-1), SBDS (CAS 831-59-4), DMBA (CAS 611-74-5), DENA (CAS 114-33-0), DMU (CAS 96-31-1), SLS (CAS 151-21-3), and PDO (CAS 63625-56-9) were purchased from Carl Roth (Steinheim, Germany). Urea (CAS 57-13-6), tween 20 (CAS 9005-64-5), tween 80 (CAS 9005-65-6), DMF (CAS 68-12-2), and DMSO (CAS 67-68-5) were purchased from Scharlau (Sentmenat, Spain). GLY (CAS 56-81-5), ethanol (64-17-5) and glacial acetic (CAS 64-19-7) acid were purchased from Chimopar (Bucharest, Romania). All substances used were of analytical reagent grade.

### 2.2. Brine Shrimp Lethality Test

*Artemia salina* cysts were hatched in beakers filled with artificial sea water at a salinity of 35 g/L, under continuous aeration, and at a temperature of 25 ± 1 °C for 48 h and a light/dark cycle of 16/8 h. The constant conditions were maintained using a plant growth chamber (Sanyo MLR-351 H, San Diego, CA, USA). After hatching, the *Artemia nauplii* were separated and brought in aerated fresh artificial sea water. A number of ten nauplii were added in tissue culture plates containing 24 wells (Greiner Bio-One, Kremsmünster, Austria), along with the tested solutions [29,30]. SXS, SBS, PTS, SBDS, DMBA, DENA, Urea, DMU, DMSO, GLY, PDO, Tween 20, Tween 80, and SLS were tested in six concentrations: 0.05, 0.1, 0.5, 1.0, 2.5, and 5.0% (*w*/*v*). Artificial sea water was used as negative control and sodium lauryl sulfate as positive control [31,32]. The artificial seawater was prepared according to the instructions provided by the *Artemia* egg manufacturer, using the supplied salts dissolved in distilled water. A total of 5 L was prepared, aerated, and stored at 4 °C, in dark for no more than two weeks. Before use, the artificial seawater was aerated again. The viability of the nauplii was checked under a stereo-microscope (STMLAB, Monza, Italy) at 24 and 48 h, the organisms being considered dead if no appendage movement was observed for at least 30 s. The lethality values were calculated and were plotted against the logarithm of concentrations, using the least square fit method to obtain lethality curves. Based on the results, the LC_50_ (lethal concentration 50%) values were calculated for each compound at the two moments of observation, and the 95% confidence interval of the LC_50_ (95% CI) was calculated using the same method. The photographs were taken using an ODC 825 Microscope Digital Camera (KERN, Balingen, Germany), with a resolution of 5.1 MP and a 1/2.5″ Aptina CMOS sensor, and processed using Microscope-VIS software 2.0 (KERN Balingen, Germany).

### 2.3. Hatching Test

Following the lethality test results, Tween 20 and Tween 80 were excluded from the HT test based on the high toxicity, whereas DMF was also excluded based on the different results obtained at the two timepoints of the assay. SLS was used as positive control and artificial sea water as negative control. The determination was performed according to the protocols described in the literature [33], with some modifications.

The *Artemia* cysts were hydrated for 60 min using artificial sea water with a salinity of 35 g/L, and approximately 20 cysts were added in 24-well plates, with concentrations in the range of 0.1 to 2.5% (*w*/*v*) of the tested substances. Each sample was performed in duplicate, and artificial sea water was used as control. The hatching process was monitored at 24 and 48 h, and stages 0 and Instar I–III were considered for the quantification. Randomization and blinding were not applied in the assays, as the evaluation of developmental stages is considered objective and unlikely to be influenced by observer bias. The inhibition of the hatching (HI) process was calculated versus the control using the formula presented by Rotini et al. [17]. HI50 was calculated by interpolating on the curves plotted, using the logarithm of concentrations and the hatching inhibition (%) with the least square fit method. Additionally, 95% confidence intervals and the correlation between concentration and inhibition were evaluated.

### 2.4. Development of Nauplii

Each sample was monitored using a stereo-microscope (STMLAB, Monza, Italy) and a bright-field microscope (Euromex, Bscope series, Groningen, The Netherlands) at both moments of determination. After 48 h, a mixture of ethanol and acetic acid in the ratio of 3:1 (*v*/*v*) was added to each sample to ensure the preservation for further observations. Depending on the development stage of each sample, the hatching process was divided into four main groups (A–D), and expressed as percents (%). The first group were the unhatched eggs (group A) that remain unchanged between the rehydration operation and the moment of observation; in the second group were the eggs with a broken shell (group B) with evident cracks and modified shape; the third category corresponds to the hatching moment, and it was noted as stage 0 (group C), when the nauplii are pear-shaped or umbrella-shaped and immobile; the fourth stage corresponds to larval stages that comprise the naupliar development stages Instar I–III (group D). All samples were compared to the untreated control to identify non-disruptive concentrations and solvent compatibility for future hatching assays [34,35].

### 2.5. Statistical Analysis

The data distribution was assessed using the D’Agostino & Pearson normality test, and due to the normality distribution, the results were analyzed statistically using one-way ANOVA, with *p* < 0.05 considered statistically significant. Post hoc comparisons were performed using Tukey’s HSD test. Results are presented as mean ± standard deviation. All calculations were carried out using GraphPad Prism v5.1 (San Diego, CA, USA) and Microsoft Excel 2021. Lethality and inhibition curves were plotted using the logarithm of the concentration versus lethality or hatching inhibition, while ANOVA and Tukey’s HSD tests were performed on the raw data. Hierarchical clustering based on Euclidean distance was applied to generate dendrograms using the Ward clustering method, also known as Ward’s minimum variance method, using SPSS Statistics v20.0 software (IBM Corp., Armonk, NY, USA).

## 3. Results

### 3.1. Lethality Test

At 24 h, the most toxic substances, causing 100% lethality at the highest concentration (5%), were SXS, PTS, SBDS, DMBA, DENA, DMU, and DMF. Among these, DMBA and DENA maintained high lethality even at lower concentrations, with DMBA showing 100% lethality down to 0.5% and minimal lethality (5–0%) at 0.1–0.05%. SBS, PTS, and SBDS also showed a concentration-dependent decrease in lethality, with SBS being less potent than PTS and SBDS at lower concentrations. Urea, DMU, and DMF demonstrated moderate toxicity at high concentrations, with reduced lethality as the concentration decreased. DMSO, GLY, and PDO showed low lethality overall, with DMSO reaching a maximum of 25% at 0.1%. SLS was consistently toxic, showing 100% lethality across all concentrations, suggesting very high acute toxicity to *Artemia*. GLY and PDO exhibited minimal lethality, with values ranging from 0 to 25%, indicating low acute toxicity at 24 h.

**Figure 1:**
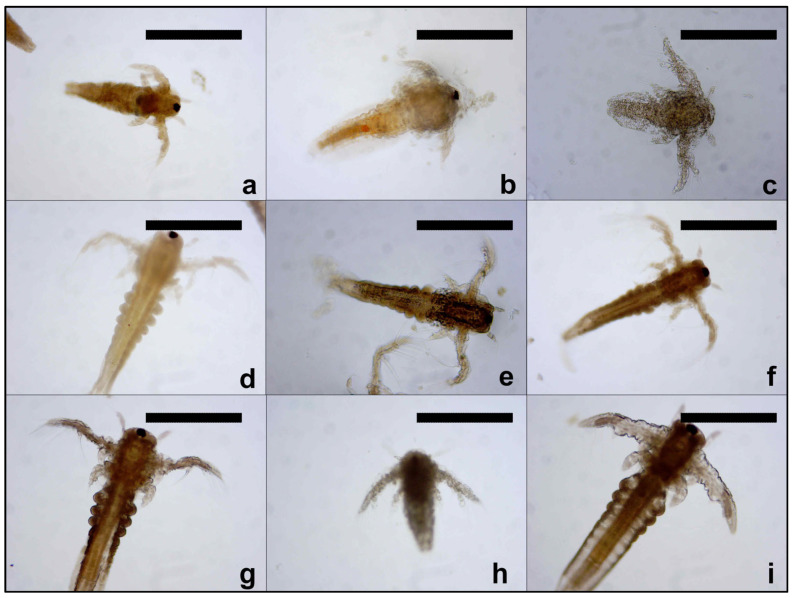
Different stages of *Artemia nauplii* after 48 h of exposure, during the lethality test: (**a**) SXS (1.0%); (**b**) SBS (0.5%); (**c**) DMBA (1.0%); (**d**) Urea (0.5%); (**e**) DMF (0.05%); (**f**) DMSO (1%); (**g**) PDO (0.05%); (**h**) DENA (2.5%); (**i**) control. Scale bars represent 500 µm.

The toxic effects were more pronounced after 48 h (Figure 1). Substances like SXS, SBS, PTS, SBDS, DMBA, DENA, and DMU maintained 100% lethality at concentrations ≥ 1%. PTS and DMBA remained highly lethal down to 0.1% (90% and 75%, respectively). DENA showed a strong time-dependent effect, with lethality increasing at lower concentrations (e.g., 45% at 0.1%). Similarly, DMU and urea showed increased lethality at 48 h, suggesting cumulative toxicity. DMF remained moderately toxic at 48 h, with up to 80% lethality at 1%, while DMSO exhibited a gradual, dose-dependent increase, peaking at 45% at 0.5%. SLS again caused 100% lethality at all concentrations, confirming its consistent and potent toxicity. GLY and PDO were among the least toxic, though GLY showed a slight increase in lethality at 48 h (up to 40%), and PDO showed 95% lethality at the highest concentration (5%), with decreasing effects at lower doses.

Thus, SLS, DMBA, PTS, DENA, and SXS could be considered highly toxic by inducing strong lethality at both timepoints and low concentrations, whereas SBS, SBDS, DMU, urea, and DMF exhibited moderate toxicity, and GLY and PDO induced low toxicity. DMSO induced moderate-to-low toxicity, with a good concentration- and time- dependent lethality on brine shrimps.

**Figure 2:**
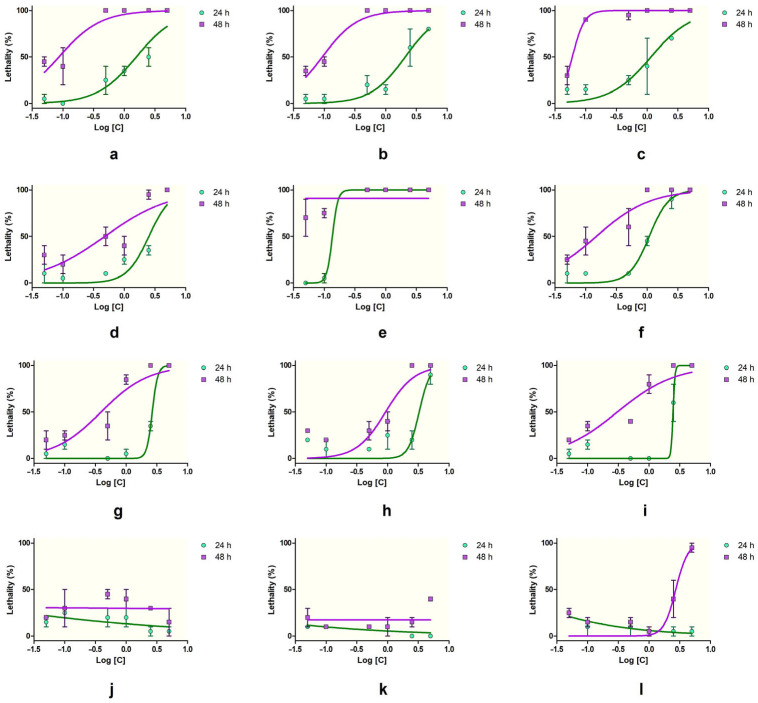
Lethality–concentration curves at 24 and 48 h for the Artemia lethality assay: (**a**) SXS, (**b**) SBS, (**c**) PTS, (**d**) SBDS, (**e**) DMBA, (**f**) DENA, (**g**) DMU, (**h**) urea, (**i**) DMF, (**j**) DMSO, (**k**) GLY, (**l**) PDO. Error bars represent the standard deviation.

The brine shrimp lethality assay revealed significant differences in acute toxicity among the tested substances, both at 24 and 48 h (ANOVA, *p* < 0.0001, Table 1 and Figure 2). At the 24 h timepoint, the lowest LC_50_ values were recorded for DMBA (0.13%), highlighting its potent lethality and the highest acute toxicity. Although the 95%CI for DMBA could not be determined due to high variability in the data, the high correlation coefficient (r^2^ = 0.998) supports the reliability of the dose–response model. Other compounds with strong toxicity at low concentrations included PTS (1.17%), DENA (0.22%), and SXS (1.01%), all of which presented relatively narrow CIs and acceptable r^2^ values (above 0.69), indicating consistent model fits. SBS (0.93%), DMF (1.24%), DMU (1.06%), SBDS (1.88%), and urea (1.11%) demonstrated moderate toxicity at 24 h, with more variable confidence intervals but overall reliable modeling.

At 48 h, an increase in toxicity was evident across nearly all compounds. The LC_50_ values decreased significantly, especially for PTS (0.28%), SXS (0.79%), and SBS (0.21%), all below 0.1%, indicating a time-dependent increase in lethality. Similarly, DENA (0.05%), DMF (1.06%), and DMU (0.08%) showed enhanced toxicity with longer exposure, and their associated confidence intervals support the robustness of these estimates. SBDS (0.14%) and urea (0.74%) maintained moderate toxicity, with LC_50_ values decreasing over time. PDO (1.64%) was the only substance classified with low toxicity for which an LC_50_ could be calculated at 48 h, further confirming its minimal lethality to *Artemia*. For several substances, LC_50_ values could not be calculated due to either extreme or minimal lethality. SLS, Tween 20, and Tween 80 caused such high mortality at all tested concentrations that LC_50_ estimation was not possible. DMSO, GLY, and PDO (at 24 h) exhibited very low toxicity, making an LC_50_ determination unable to be determined due to insufficient mortality.

The highest acute toxicity was associated with DMBA, PTS, SXS, and SBS, all showing LC_50_ values of ≤1% at both timepoints. Moderate toxicity was observed for DENA, DMU, SBDS, urea, and DMF, with LC_50_ values generally ranging between 0.3% and 3%. Low-toxicity compounds included PDO, DMSO, and GLY.

**Figure 3:**
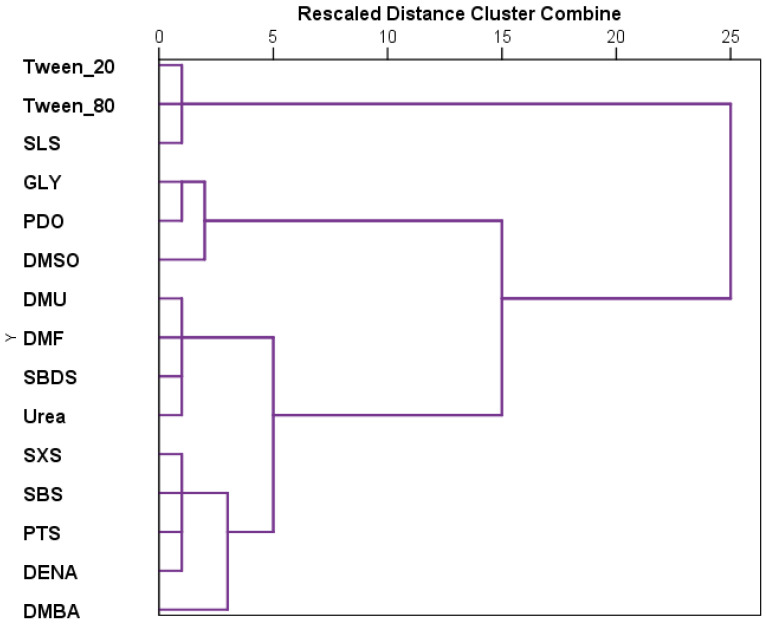
Dendrogram cluster of the tested substances based on the lethality induced on *Artemia salina* nauplii. The axis represents the rescaled distances between clusters, based on the minimum and the maximum distances in the proximity matrix.

The results recorded for each substance were further analyzed using hierarchical cluster analysis (Figure 3). The tested substances can be grouped in three main clusters, the last one presenting two other clusters. Thus, the first cluster includes the most toxic compounds, which are tween 20, tween 80, and SLS. The second cluster is the grouping GLY, PDO, and DMSO, and the last cluster the remaining substances. The two secondary clusters that derive from the third one are represented on one side by sulfonate derivatives along with DENA and DMBA, and on the other, by DMU, urea, SBDS, and DMF.

### 3.2. Hatching Inhibition

The tested substances exhibited different inhibitory effects compared to the untreated control and could be grouped depending on this criterion into relatively low to comparable to control, low moderate, and strong inhibitory effect. Also, between the moments of determination, belonging to a certain group of toxicity changed.

The inhibition of hatching varied across substances and concentrations, with some compounds showing strong suppression of development, while others had minimal or no effect. SLS caused complete or near complete inhibition at all concentrations and timepoints, confirming its potent embryotoxicity. SXS followed a similar pattern, reaching 100% inhibition at 2.5% and showing strong dose-dependent effects from 0.5% upward. DMBA also demonstrated significant inhibition, especially at 24 h, with values exceeding 90% at high concentrations. Although inhibition decreased slightly at 48 h, it remained significant. DENA showed full inhibition at 2.5% but minimal effects at lower doses, indicating a clear threshold-dependent toxicity. PTS showed variable inhibition, with strong suppression at 2.5%, but inconsistent results at lower concentrations. SBDS, DMU, and urea caused mild-to-moderate inhibition, generally remaining under 50%, with slight reductions over time. DMSO, PDO, and GLY showed very low or no inhibition. Some negative values suggest no hatching interference and possible variability. SBS also caused only moderate suppression, particularly at 48 h.

**Figure 4:**
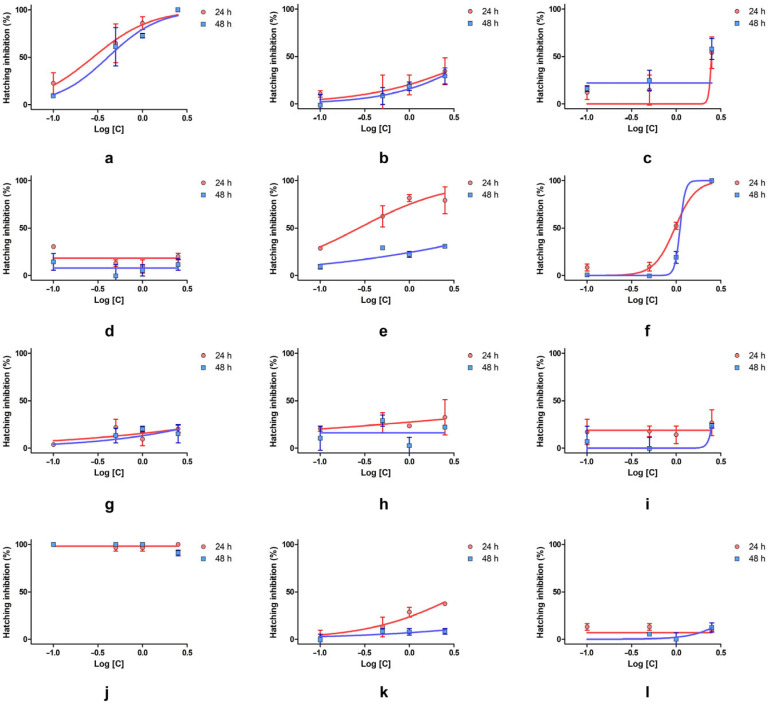
Inhibition–concentration curves at 24 and 48 h for the Hatching inhibition test: (**a**) SXS, (**b**) SBS, (**c**) PTS, (**d**) 0 SBDS, (**e**) DMBA, (**f**) DENA, (**g**) DMU, (**h**) urea, (**i**) DMSO, (**j**) SLS, (**k**) GLY, (**l**) PDO. Error bars represent the standard deviation.

Among the tested substances, only a few allowed for IC_50_ determination due to the nature of the response curves (Table 2 and Figure 4). SXS showed consistently high inhibition, with IC_50_ values of 0.28% (24 h) and 0.41% (48 h). The relatively narrow confidence intervals and solid r^2^ values indicate a good concentration–response relationship. These results confirm SXS as a strong inhibitor of hatching. DMBA also exhibited strong inhibition at 24 h, with an IC_50_ of 0.27% and a good model fit (r^2^ = 0.79). However, at 48 h, the IC_50_ value was anomalously high (19.23%) and lacked a defined confidence interval, suggesting weaker or variable inhibition over time. DENA allowed for IC_50_ estimation at both timepoints, with values of 0.97% (24 h) and 1.10% (48 h). The narrow confidence interval at 24 h and excellent fit (r^2^ = 0.98) suggest a consistent and moderate inhibitory effect, with a slight decrease in potency over time. PTS induced a IC_50_ of 2.49% at 24 h, but the fit was weaker (r^2^ = 0.59), and no values could be determined at 48 h. This points to low and inconsistent inhibition. For the other compounds, including SBS, SBDS, DMU, urea, DMSO, SLS, GLY, and PDO, IC_50_ values could not be determined due to either very low or extremely high inhibition across all concentrations.

ANOVA revealed statistically significant differences at both the 24 h and 48 h timepoints, with *p* < 0.0001 in each case.

### 3.3. Hatching and the Development of Nauplii

**Figure 5:**
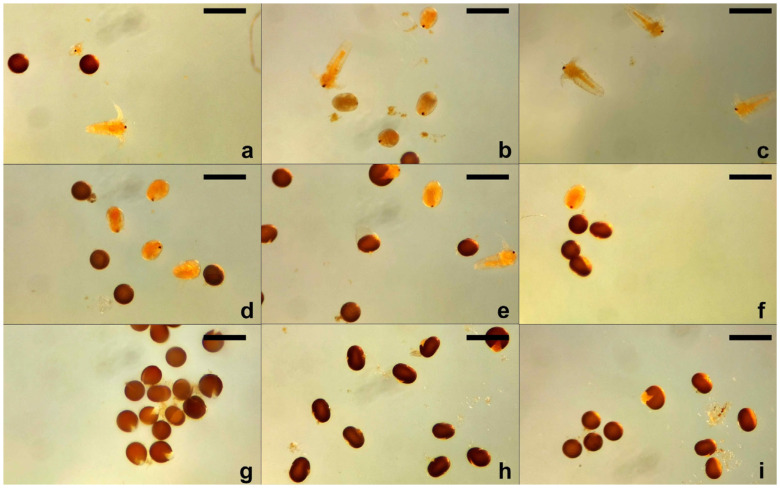
Different stages of hatching after exposure to the testing compounds: unhatched eggs (**a**,**d**,**f**,**g**); cysts with broken shell (**e**,**f**,**g**,**h**,**i**); nauplii with pear shape or umbrella shape and immobile (**b**,**d**,**e**,**f**); naupliar development stages (**a**,**b**,**c**,**e**). Scale bars represent 500 µm.

**Figure 6:**
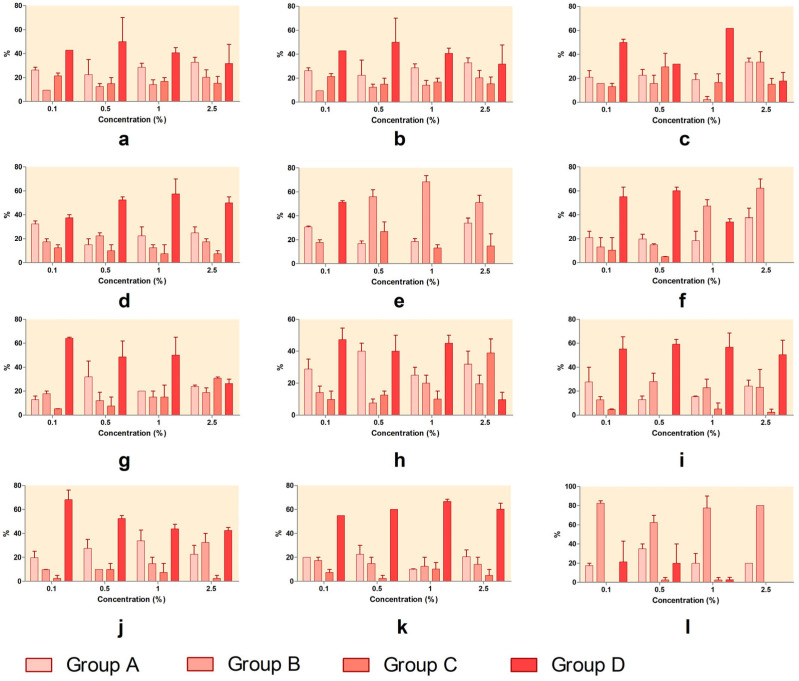
Histograms of the hatching process at 24 h of exposure across the four groups: A—unhatched eggs that remain unchanged between the rehydration operation and the moment of observation; B—eggs with broken shell with evident cracks and modified shape; C—the hatching moment, stage 0, when the nauplii are pear-shaped or umbrella-shaped and immobile; D—larval stages that comprise the naupliar development stages Instar I–III. Tested compounds: (**a**) SXS, (**b**) SBS, (**c**) PTS, (**d**) SBDS, (**e**) DMBA, (**f**) DENA, (**g**) DMU, (**h**) urea, (**i**) DMSO, (**j**) SLS, (**k**) GLY, (**l**) PDO. Error bars represent the standard deviation.

**Figure 7:**
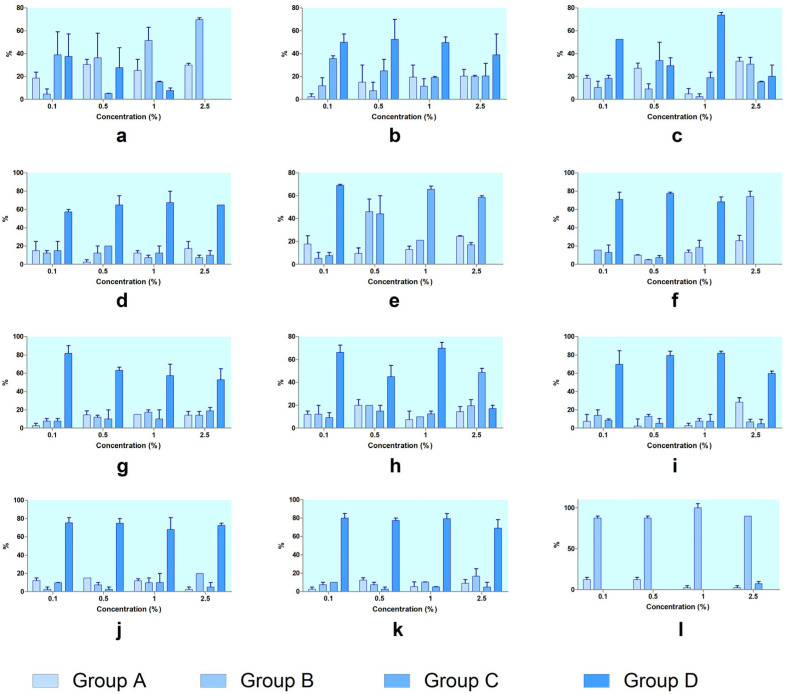
Histograms of the hatching process at 48 h of exposure across the four groups: A—unhatched eggs that remain unchanged between the rehydration operation and the moment of observation; B—eggs with broken shell with evident cracks and modified shape; C—the hatching moment, stage 0, when the nauplii are pear-shaped or umbrella-shaped and immobile; D—larval stages that comprise the naupliar development stages Instar I–III. Tested compounds: (**a**) SXS, (**b**) SBS, (**c**) PTS, (**d**) SBDS, (**e**) DMBA, (**f**) DENA, (**g**) DMU, (**h**) urea, (**i**) DMSO, (**j**) SLS, (**k**) GLY, (**l**) PDO. Error bars represent the standard deviation.

The hatching progression of *Artemia* cysts was evaluated by analyzing their distribution across developmental stages at each concentration of the tested compounds (Figure 5). In the control group, *Artemia* showed normal development, with the majority progressing to Group D. At 24 h, approximately 72% had reached Group C, with nearly 60% in Group D. By 48 h, over 84% had fully developed into Group D, confirming healthy baseline conditions. Upon exposure to the tested substances, distinct patterns emerged and are presented in Figure 6 (at 24 h of exposure) and Figure 7 (at 48 h of exposure). Thus, SXS caused a clear developmental block. As the concentration increased, the proportion of individuals in Group A and Group B rose, while Group C and Group D declined. At 2.5%, no development beyond Group B occurred, both at 24 h and 48 h, indicating strong inhibition of hatching and post-hatching progression. SBS produced a moderate developmental delay. Although Groups A and B increased with concentration, a large proportion of larvae still reached Group D, especially at 48 h. This suggests partial interference with development but not complete arrest. PTS allowed considerable development at lower concentrations. At 1%, over 60% reached Group D at 48 h. However, at 2.5%, Groups A and B became dominant, with a decline in hatched stages, indicating dose-dependent developmental effects. SBDS showed limited disruption. At both timepoints and all concentrations, a large percentage of individuals reached Group D, demonstrating that SBDS had a low impact on embryonic development.

DMBA had a pronounced effect on development. From 0.5% upward, most individuals remained in Group A or Group B, with no significant presence in Group D, confirming severe interference with both the hatching and post-hatching stages.

DENA showed a shift in developmental outcome depending on concentration. At low doses, most embryos progressed to Group D, but at 2.5%, development was arrested in Groups A and B, indicating strong toxicity at higher concentrations. DMU permitted development at low concentrations, especially at 48 h, where over 80% of individuals reached Group D at 0.1%. However, increased doses led to rising proportions in Groups A and B, showing moderate dose-dependent effects. Urea had a similar profile to DMU. At lower concentrations, development to Group D was common, particularly at 48 h. At 2.5%, however, more individuals remained in Groups A and B, with a reduction in Group D, suggesting some developmental delay. DMSO showed very low developmental toxicity. Most individuals successfully reached Group D at all concentrations and timepoints, with minimal shifts toward earlier stages. Even at 2.5%, over 80% of embryos developed fully, confirming its safety profile. SLS caused complete developmental arrest. Regardless of concentration, nearly all individuals remained in Group A or Group B, with no progression to Groups C or D, indicating very strong hatching inhibition and embryotoxicity. GLY had a negligible impact on development. Most embryos developed to Group D, especially at 48 h, with only minor accumulation in earlier stages, even at higher concentrations. PDO also showed minimal interference. Across all concentrations, a high proportion of individuals reached Group D, particularly after 48 h, confirming PDO as a low-toxicity compound in terms of *Artemia* development (Figure 7).

**Figure 8:**
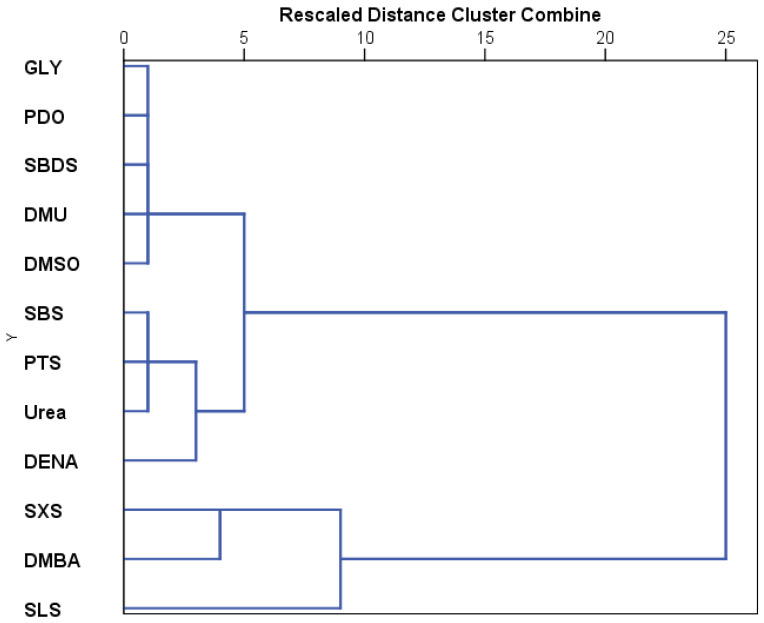
Dendrogram cluster of the tested substances based on the influence on the development of *Artemia salina.* The axis represents the rescaled distances between clusters, based on the minimum and the maximum distances in the proximity matrix.

Based on the stage development under the influence of the tested substances, the hierarchical cluster analysis indicates three major clusters (Figure 8). The first cluster represents SLS, the second includes DMBA and SXS, and the third cluster includes the other compounds tested. The third cluster can be further divided into two other clusters, one that contains DENA, urea, SBS, and PTS, and the other that groups GLY, PDO, DMSO, SBDS, and DMU.

## 4. Discussion

This study evaluated the acute and developmental toxicity of selected hydrotropes and co-solvents using two complementary bioassays—lethality and hatching inhibition—in *Artemia salina*. By comparing multiple endpoints, including mortality, hatching success, and developmental stage distribution, we aimed to distinguish compounds with high toxic potential from those suitable for use in biological formulations. To guide interpretation and highlight key findings, the discussion is structured around specific questions.

What was the rationale and approach of this study? In the present study, two bioassay methods were applied to a selection of chemicals, including hydrotropes and co-solvents commonly used in the pharmaceutical and cosmetic industries, as well as in various research fields. *Artemia* species are halophilic crustaceans widely employed to evaluate the toxicity of newly synthesized compounds, pharmaceuticals, environmental pollutants, wastewater, and plant extracts [12,15,36].

Currently, two species of the *Artemia* genus are predominantly used in toxicity testing: *Artemia salina* and *Artemia franciscana* [37,38,39]. A key advantage of these species is their ability to rapidly hatch from dormant cysts upon rehydration, enabling a fast and efficient setup for experimental assays. This biological feature facilitates convenient, on demand access to larvae, contributing to their popularity in ecotoxicological research.

In this study, two complementary bioassays—lethality and hatching inhibition—were applied to assess the acute toxicity and embryotoxicity of several compounds commonly used in pharmaceutical and cosmetic industries. The tested substances were evaluated using *Artemia salina*, a widely accepted model organism for toxicity screening.

Which substances exhibited the highest toxicity and how? The results showed that lethality testing provides a rapid and practical method to assess acute toxicity, with the ability to process a large number of compounds in a short period. The 24 h lethality results indicated that SXS, PTS, SBDS, DMBA, DENA, DMU, and DMF induced complete mortality at 5%, while SLS caused 100% lethality across all concentrations. As expected, the toxic effects were even more pronounced at 48 h, with most of these compounds maintaining full lethality at ≥1%. Substances like DMBA and DENA showed high toxicity even at low concentrations, indicating strong time- and dose-dependent effects.

GLY, PDO, and DMSO exhibited low acute toxicity, with minimal lethality at both timepoints, results that sustain the wide use in brine shrimp lethality tests for assessing the toxicity of plant extracts and chemical compounds [40,41]. SLS, DMBA, PTS, DENA, and SXS could be classified as highly toxic based on their ability to induce strong lethality even at low concentrations and short exposures. SBS, SBDS, DMU, urea, and DMF showed moderate toxicity, while DMSO, PDO, and GLY were consistently among the least toxic. The results are in accordance with the wide use of these three compounds in small concentrations to enhance the solubility of potential bioactive compounds or plant extracts [18,40,42].

LC_50_ values provided quantitative support for these trends. At 24 h, the lowest LC_50_ values were observed for DMBA (0.13%), PTS (1.17%), DENA (0.22%), and SXS (1.01%), with a further decrease at 48 h for PTS (0.28%), SXS (0.79%), and DENA (0.05%), confirming a time-dependent increase in toxicity. Compounds such as SBS, DMF, DMU, SBDS, and urea had LC_50_ values in the moderate range (0.3–3%), while PDO was the only low-toxicity compound with a measurable LC_50_ at 48 h. For SLS, Tween 20, and Tween 80, lethality was too high to allow LC_50_ estimation, while DMSO, GLY, and PDO (at 24 h) showed lethality too low to permit calculation. The results are in line with other researchers’ findings [43].

Despite the advantages of lethality testing, particularly its speed and scalability, the observations highlight several important limitations [17]. First, hatched, mobile larvae tend to be more resistant to the tested compounds, potentially masking early embryotoxic effects. Second, the variability in developmental stages introduces biological heterogeneity, which can affect reproducibility. However, this variability is not necessarily a drawback, as it can also reflect natural population dynamics. Nevertheless, lethality testing alone cannot evaluate embryotoxicity, a limitation that is particularly relevant when screening compounds that interfere with early development.

To address this, the hatching inhibition test was employed, offering greater sensitivity in detecting developmental modifications [17,33]. The test revealed that substances such as SLS, SXS, and DMBA significantly inhibited hatching at nearly all concentrations and timepoints, confirming their strong embryotoxic potential. DENA showed full inhibition at 2.5% but negligible effects at lower doses, indicating a sharp toxicity threshold. GLY, PDO, and DMSO caused minimal or no inhibition, consistent with their low-toxicity profiles in the lethality assay.

Hatching inhibition allows direct observation of nauplii development under chemical exposure, eliminating the confounding effect of larval mobility [44]. It also enables the assessment of embryotoxicity, which lethality tests cannot capture [45]. However, the method is more time-consuming and less suited to high-throughput applications.

When IC_50_ values were calculated for hatching inhibition, only SXS, DMBA, and DENA yielded reliable values at both timepoints, confirming their strong dose–response relationships. For most other substances, IC_50_ estimation was not feasible due to either very low or very high inhibition rates.

The results from developmental stage distribution analyses further support the hatching inhibition data. In the untreated control, most individuals reached Group D (fully developed nauplii) by 48 h, while substances like SLS, SXS, and DMBA caused marked developmental arrest, with embryos remaining in Groups A or B (unhatched or broken chorion). Substances like PTS, DENA, DMU, and urea showed dose-dependent developmental delays, while GLY, PDO, DMSO, and SBDS allowed normal progression to Group D, even at higher concentrations.

What mechanisms may explain the observed developmental toxicity? The strong embryotoxicity observed with DMBA, SLS, and SXS likely results from different underlying mechanisms. DMBA, a small aliphatic amine, may disrupt membrane integrity or interfere with cell signaling pathways critical for morphogenesis. SLS, a known surfactant, is capable of denaturing proteins and disrupting lipid membranes, which can compromise early cell division and gastrulation. SXS, a sulfonated aromatic compound, may exert toxicity by interfering with energy metabolism or by acting as a membrane destabilizer. These mechanisms align with findings in other aquatic toxicity models and raise concerns regarding the embryotoxic potential of these substances in broader biological systems [43,44,45].

Hierarchical clustering of stage development patterns revealed three major clusters. The most toxic group—SLS, Tween 20, and Tween 80—formed the first cluster. A second cluster grouped GLY, PDO, and DMSO, representing the least toxic substances. The remaining substances formed a third cluster, which could be further subdivided into (1) sulfonate derivatives with DENA and DMBA, and (2) DMU, urea, SBDS, and DMF.

What are the practical implications of these findings? The results suggest that compounds such as DMSO, PDO, and GLY, which showed minimal lethality and embryotoxicity, may be more suitable as solvents or carriers in formulations intended for biological applications. In contrast, substances like SLS, DMBA, and SXS should be used with caution, particularly in scenarios involving aquatic discharge or developmental exposure. A practical threshold based on this study would be to avoid concentrations ≥ 1% for compounds showing LC_50_ or full inhibition at or below this level, especially in early-life-stage assays.

What limitations exist, and how can future studies build on this work? Although the hatching inhibition test offers greater sensitivity to embryotoxic effects, its labor-intensive nature limits high-throughput use. Furthermore, in vitro tests do not account for metabolic transformation or organism-level compensatory mechanisms. Future work could involve transcriptomic or proteomic profiling to elucidate pathways affected by embryotoxic compounds and validate results in more complex organisms.

## 5. Conclusions

Combining both lethality and hatching inhibition tests provided a comprehensive overview of the toxicological profiles of various hydrotropes and co-solvents commonly used in the pharmaceutical and cosmetic industries. While lethality assays are efficient for screening acute toxicity, the hatching test offers greater sensitivity to early developmental effects and embryotoxicity, particularly in species like *A. salina*, where asynchronous hatching introduces biological variability.

Despite its labor-intensive nature, the hatching test proved essential for detecting subtle developmental disruptions and for distinguishing between compounds that are lethally toxic and those that interfere primarily with embryogenesis. Compounds such as SLS, SXS, and DMBA were consistently associated with high lethality and significant hatching inhibition, even at low concentrations. In contrast, DMSO, GLY, and PDO showed minimal toxicity in both assays, supporting their continued use as safer solvents or excipients. From a practical perspective, the data support the following recommendations: (1) Avoid concentrations ≥ 1% for compounds that exhibit LC_50_ or full hatching inhibition at or below this threshold (e.g., SLS, DMBA, SXS), particularly in formulations with potential environmental discharge or developmental exposure. (2) Prefer solvents like DMSO, GLY, and PDO in hatching-based ecotoxicological assays or bioactive compound delivery, given their low impact on both survival and development. (3) Incorporate hatching inhibition assays alongside lethality testing in early-stage screening, especially when evaluating novel compounds for pharmaceutical or cosmetic applications.

These findings contribute to the development of more refined safety thresholds and reinforce the need for integrating embryotoxicity endpoints into routine toxicological assessments, especially for compounds intended for human use or likely to enter aquatic environments.

## Figures and Tables

**Table 1:** Results of brine shrimp lethality assay.

	LC_50_		95%CI of LC_50_		r^2^	
	(%)		(%)			
Substance	24 h	48 h	24 h	48 h	24 h	48 h
SXS	1.01	0.79	0.93–1.07	0.63–0.98	0.8286	0.9315
SBS	0.93	0.21	0.53–1.59	0.11–0.44	0.7694	0.7671
PTS	1.17	0.28	0.78–1.74	0.18–0.42	0.9254	0.9274
SBDS	1.88	0.14	ND	0.09–0.18	0.998	0.9434
DMBA	0.13	0.09	ND	ND	0.998	ND
DENA	0.22	0.05	0.18–0.25	0.04–0.06	0.9912	0.9704
DMU	0.94	0.08	0.71–1.25	0.06–0.10	0.8704	0.93
Urea	1.11	0.74	ND	0.57–1.52	0.9981	0.9096
DMF	1.24	1.06	ND	0.81–1.37	0.9094	0.9248
DMSO	ND **	ND **	ND	ND	ND	ND
Tween 20	ND *	ND *	ND	ND	ND	ND
Tween 80	ND *	ND *	ND	ND	ND	ND
SLS	ND *	ND *	ND	ND	ND	ND
GLY	3.72	ND **	ND	ND	ND	ND
PDO	ND	1.64	ND	0.94–2.84	0.6901	0.7745

LC_50_—50% lethal concentration; 95%CI—95% confidence interval; ND—not determined due to the obtained results; *—the lethality values were too high to calculate LC_50_; **—the lethality values were too low to calculate LC_50_.

**Table 2:** Results of *Artemia salina* cysts hatching inhibition.

Substance	IC_50_		95%CI of IC_50_		r^2^	
	(%)		(%)			
	24 h	48 h	24 h	48 h	24 h	48 h
SXS	0.28	0.41	0.14–0.54	0.24–0.69	0.841	0.8925
SBS	ND *	ND *	ND **	ND **	0.3042	0.6153
PTS	~2.49	ND ***	ND **	ND **	0.5938	ND **
SBDS	ND *	ND *	ND **	ND **	ND **	ND **
DMBA	0.27	19.23	0.13–0.55	ND **	0.7943	0.7456
DENA	0.97	1.10	0.86–1.1	ND **	0.9775	0.9942
DMU	ND *	ND *	ND **	ND **	ND **	ND **
Urea	ND *	ND *	ND **	ND **	ND **	ND **
DMSO	ND *	ND *	ND **	ND **	ND **	ND **
SLS	ND ***	ND ***	ND **	ND **	ND **	ND **
GLY	ND *	ND *	ND **	ND **	ND **	ND **
PDO	ND *	ND *	ND **	ND **	ND **	ND **

IC_50_—50% inhibition concentration; 95%CI—95% confidence interval; ND—not determined due to the obtained results; *—the lethality values were too high to calculate IC_50_; **—the inhibition values were too low to calculate IC_50_; ***—95%CI is very wide.

## Data Availability

The original contributions presented in this study are included in the article. Further inquiries can be directed to the corresponding authors.
